# Supplementary material for: Criticality and increased intrinsic neural timescales in stroke
Source: NPJ Syst Biol Appl. 2025 Dec 7;12:4. doi: 10.1038/s41540-025-00626-7 (PMC12770600; doi:10.1038/s41540-025-00626-7)
Supplement: Supplementary file 1 — Supplementary Information [file 41540_2025_626_MOESM1_ESM.pdf]

Supplementary Table I – Demographics and clinical characteristics of participants.

Patient	Sex	Age	Side of Lesion	Lesion location	Lesion Volumes (CM^3)	Artery	Day Since Stroke	Timing BS PAT1	PAT2	PAT3	PAT4	PAT5	Recovery
Good recovery group													
P2	F	65	R	Cerebellum	0.91	PICA/SCA	26	2	4	5	6	6	good
P3	M	54	L	Thalamus	3.61	Thalamic perforators	42	1	2	3	3	3	good
P4	M	58	L	Temporal lobe/basal ganglia	1.53	MCA	22	4	5	6	6	6	good
P11	F	78	R	Posterior limb of internal capsule	2.39	MCA	22	1	1	3	3	3	good
P13	M	47	L	Precentral/Mid frontal	6.23	MCA	18	3	4	5	5	5	good
P14	M	57	L	Pallidum	6.44	Pallidum	18	1	3	3	4	4	good
Mean±SD	-	*59.42±9.05*	-	-	*3.93±2.24*		*-24.66±8.21*	*2±1.06*	*3.16±1.34*	*4.16±1.21*	*4.50±1.25*	*4.50±1.25*	good
Poor recovery group													
P1	M	59	R	Temporal/occipit lobe	63.55	PCA	16	5	5	5	6	6	poor
P5	M	63	R	Thalamus/Hippcocampus	8.68	Thalamic perforators	20	3	2	2	3	4	poor
P6	F	67	R	Thalamus/cuneus/lingual	39.81	Thalamic perforators	23	1	1	1	1	1	poor
P7	F	48	L	Thalamus	0.95	Thalamic perforators	21	6	6	6	6	6	poor
P8	M	48	L	Pallidum	1.09	Pallidum	24	6	6	6	6	6	poor
P9	M	80	L	Mid Occipital/lingual	50.51	PCA	26	1	1	2	2	2	poor
P10	M	78	L	Thalamus	3.12	Thalamic perforators	27	6	6	6	6	6	poor
P12	M	81	L	Hippocampus	1.04	PCA	27	1	1	1	1	1	poor
P15	M	73	L	Putamen	5.33	Pallidum	14	6	6	6	6	6	poor
Mean±SD	-	*66.33±12.11*	-	-	*19.43±19.54*	-	*22±4.42*	*3.88±2.23*	*3.77±2.29*	*3.88±2.21*	*4.11±2.18*	*4.11±2.28*	poor

Abbreviations: R: Right, L: Left, BS: Brunnstrom stage, SD: Standard deviation.PAT1: 30-40 days, PAT2: 60-70 days, PAT3: 90-100 days, PAT4: 120-130 days. PAT5: 150-170 days.
MCA: Middle cerebral artery. PCA: Posterior Cerebral Artery. PICA: Posterior Inferior Cerebellar Artery. SCA: Superior Cerebellar Artery.

Supplementary Table II – The parcellation encompasses eight large-scale functional networks, comprising a total of 32 regions of interest (ROIs).

#	Network	ROI	Coordinates	#	Network	ROI	Coordinates
1	Default mode network (DMN)	Medial prefrontal cortex (MPFC)	1,55,-3	2	Visual network (VIS)	Medial	2,-79,12
		Precuneus cortex (PCC)	-39,-77,33			Occipital	0,-93,-4
		Lateral parietal (LP) (Right)	47,-67,29			Bilateral (Right)	-37,-79,10
		Lateral parietal (LP) (Left)	47,-67,29			Bilateral (Left)	38,-72,13
3	Salience network (SAN)	Anterior cingulate cortex (ACC)	0,22,35	4	Sensorimotor network (SMN)	Superior	-55,-12,29
		Anterior insula (AI) (Right)	-44,13,1			Bilateral (Right)	56,-10,29
		Anterior insula (AI) (Left)	47,14,0			Bilateral (Left)	0,-31,67
		Borstal prefrontal cortex (RPFC) (Right)	-32,45,27	5	Dorsal attention network (DAN)	bilateral frontal eye field (FEF)	-27,-9,64
		Rostral prefrontal cortex (RPFC) (Left)	32,46,27			bilateral frontal eye field (FEF)	30,-6,64
		Supramarginal gyrus (SMG) (Right)	-60,-39,31			intraparietal sulcus (IPS)	-39,-43,52
		Supramarginal gyrus (SMG) (Left)	62,-35,32			intraparietal sulcus (IPS)	39,-42,54
6	Frontoparietal network (FPN)	Posterior parietal cortex (PPC) (Right)	52,-52,45	7	Language network(LN)	Inferior frontal gyrus (Right)	54,28,1
		Lateral prefrontal cortex (LPFC) (Right)	41,38,30			Inferior frontal gyrus (IFG) (Left)	-51,26,2
		Lateral prefrontal cortex (LPFC)	-43,33,28			Posterior superior temporal gyrus (pSTG) (Right)	-57,-47,15
		Posterior parietal cortex (PPC) (Left)	-46,-58,49			Posterior superior temporal gyrus (pSTG) (Left)	59,-42,13
8	Cerebellar network (CE)	Anterior	0,-63,-30	-	-	Posterior	0,-79,-32

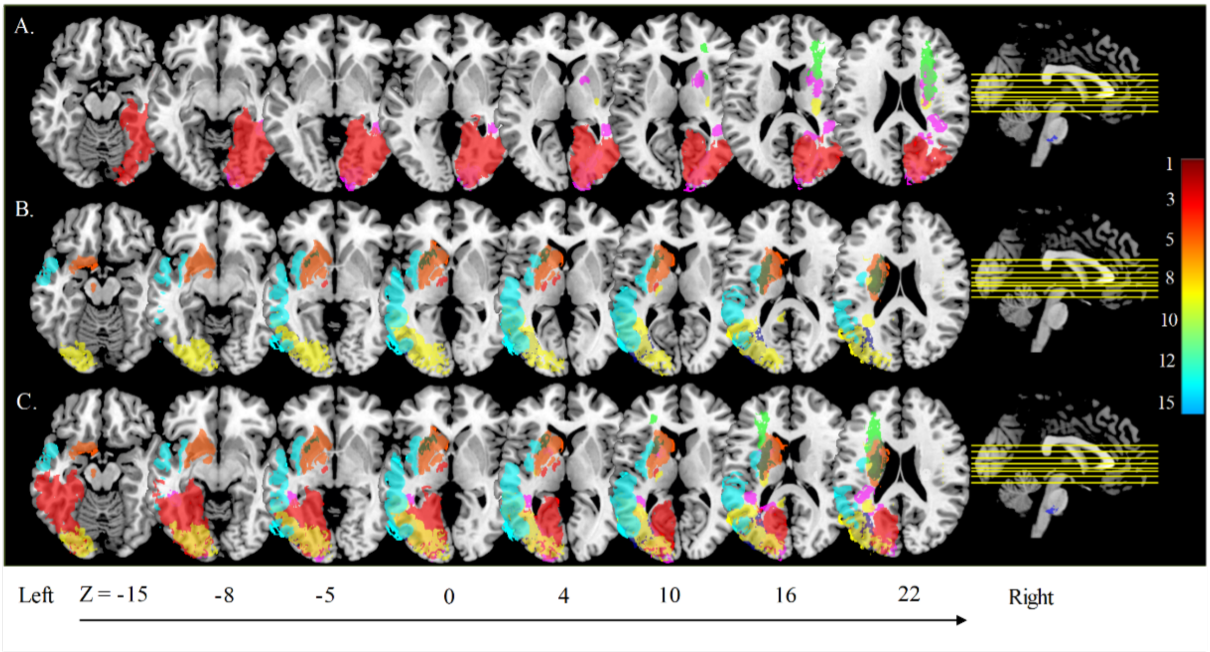

Supplementary Figure 1 – Lesion overlap map across 15 stroke patients. **(A)** right-sided lesion (n = 5); **(B)** left-sided lesion (n = 10); **(C)** the entire sample of stroke patients with right-sided lesion flipped to left-sided (n = 15). The color bar indicates the patients. See supplementary table 1 for additional details.

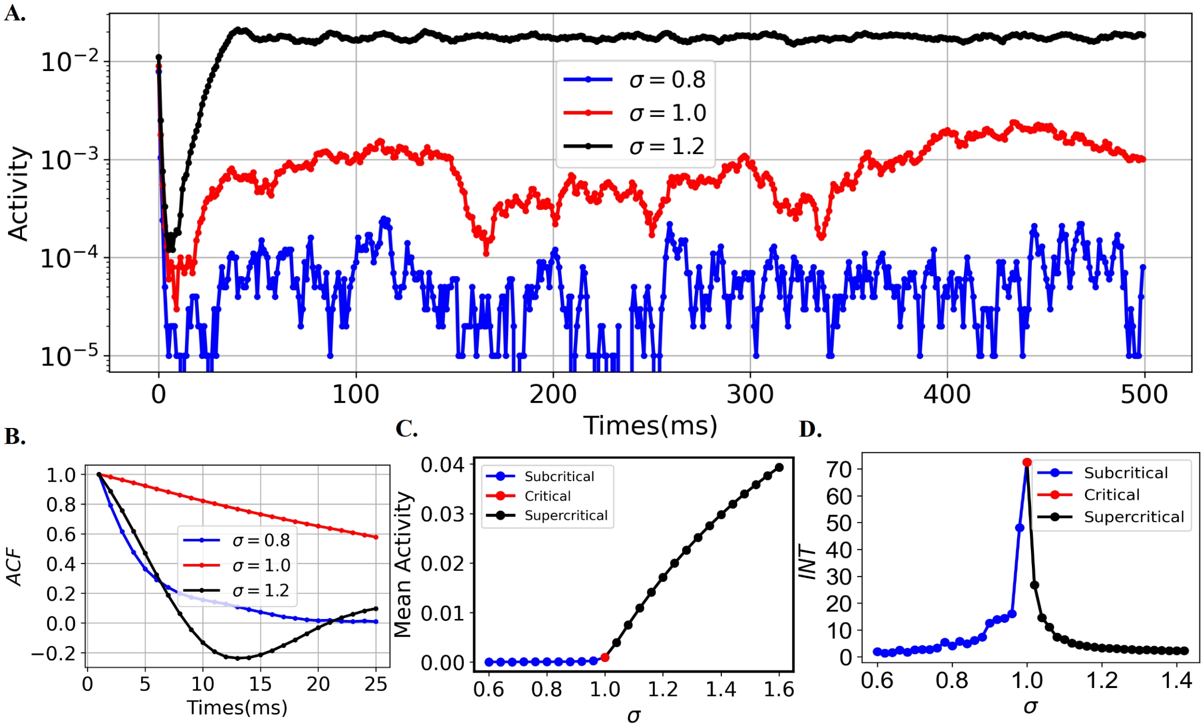

Supplementary Figure 2 – Neuronal network model dynamics. (A) Density of active neurons as a function of time for one trial with branching ratio $\sigma = 0.8$, 1.0, and 1.2. The activity is color coded with blue indicating the subcritical regime, red indicating the critical state, and black indicating the supercritical regime. **(B)** Exemplar autocorrelation function for branching ratios $\lambda = 0.8, 1.0$ and 1.2. **C.** Mean activity as a function of branching ratio. There is a phase transition at $\sigma = 1$. **D.** Relationship between intrinsic timescale and branching ratio for a single trial. All networks have N = 100,000 neurons and a mean degree $K = 10$ with varied values of λ to satisfy the relationship $\sigma = K * \lambda$. The external driving is given by $r = 10^{-5}$, see methods for details.

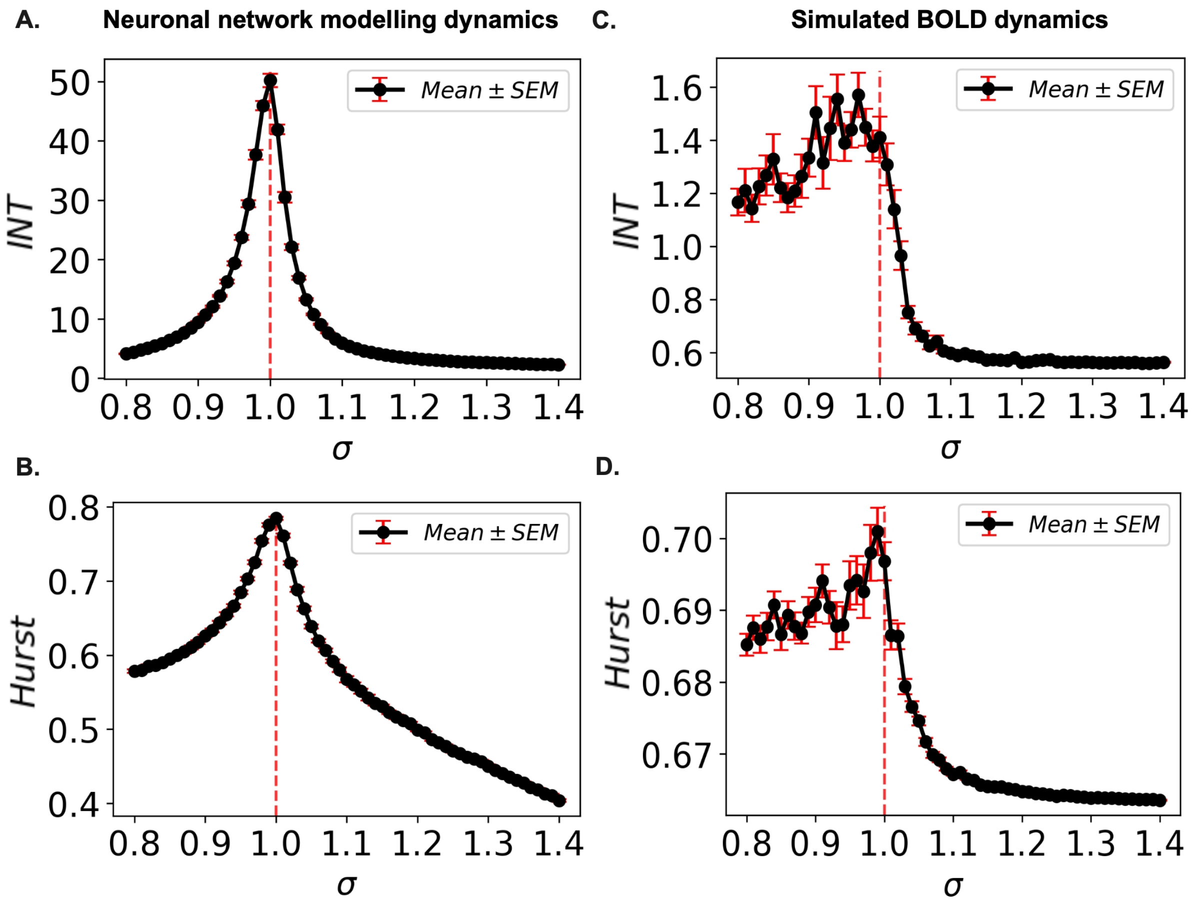

Supplementary Figure 3 – Signatures of criticality in long simulated neuronal and blood oxygenation level-dependent time series. Intrinsic neural timescales (INT, **A**, **C**) and Hurst exponents (**B**, **D**) obtained from long simulated time series. The left panels show metrics calculated from 300s of direct neuronal network activity. The right panels show the same metrics from a simulated BOLD signal, generated by convolving the neural activity with a canonical hemodynamic response function (HRF; TR = 2s, 150 timepoints). Results are averaged over 50 trials. Although the HRF convolution attenuates the signal and increases noise, a distinct peak near criticality ($\sigma = 1$) persists in both the neural and BOLD-derived measures.
